# Características histológicas de la dentina bovina mediante tinción Tricrómica de Masson

**DOI:** 10.21142/2523-2754-1104-2023-176

**Published:** 2023-12-28

**Authors:** Melisa Raquel Lezcano, Nathalie Enz, María Constanza Affur, María Alejandra Gili

**Affiliations:** 1 Division of orthodontic, Universidad Cientifica del Sur. Lima, Peru. mlezcano@odn.unne.edu.ar, nenz@odn.unne.edu.ar, mcaffur@odn.unne.edu.ar, magili@odn.unne.edu.ar Universidad Científica del Sur Division of orthodontic Universidad Cientifica del Sur Lima Peru mlezcano@odn.unne.edu.ar nenz@odn.unne.edu.ar mcaffur@odn.unne.edu.ar magili@odn.unne.edu.ar

**Keywords:** dentina, diente bovino, descalcificación, tricrómica de Masson, dentin, bovine tooth, decalcification, Masson's trichrome

## Abstract

**Objetivos:**

Identificar las características histológicas de la dentina bovina mediante técnica histológica por descalcificación con coloración tricrómica de Masson.

**Materiales y métodos:**

Estudio de tipo observacional y descriptivo con uso de unidades de análisis. Se emplearon piezas dentarias bovinas, las cuales fueron sometidas a descalcificación y coloración tricrómica de Masson. Se realizó la observación microscópica y el registro de las estructuras.

**Resultados:**

En la observación microscópica de los dientes sometidos a descalcificación se observó dentina, predentina y la organización estructural de la dentina tubular y peritubular. Los odontoblastos se mostraron dispuestos en empalizada, con la prolongación odontoblástica incluida dentro del túbulo dentinario y el espacio periprocesal. No se observa la presencia de dentina interglobular en zonas de dentina coronaria. El patrón histomorfológico correspondiente a dentina-predentina-pulpa se encuentra dispuesto de forma similar a los dientes humanos.

**Conclusiones:**

La dentina bovina constituye un sustrato ideal para trabajos de investigación in vitro con biomateriales odontológicos, ya que presenta características histológicas similares a los dientes bovinos. La ausencia de dentina interglobular no representaría una diferencia significativa, según lo observado en este estudio.

## Abstract (en)

**Objectives:**

Identify the histological characteristics of bovine dentin using histological technique by decalcification with Masson's Trichrome staining.

**Materials and Methods:**

This was an observational and descriptive study using units of analysis. Bovine teeth were used, which were subjected to a decalcification technique and Masson's trichrome staining. Microscopic observation and registration of the structures was carried out.

**Results:**

In the microscopic observation of the teeth subjected to decalcification, dentin, predentin and the structural organization of the tubular and peritubular dentin were observed. The odontoblasts arranged in a palisade with the odontoblastic process included within the dentinal tubule and the periprocedural space. The presence of interglobular dentin is not observed in areas of coronary dentin. The histomorphological pattern corresponding to dentin-predentin-pulp is arranged in a similar way to human teeth.

**Conclusions:**

Bovine dentin constitutes an ideal substrate for in vitro research work with dental biomaterials, since it presents histological characteristics similar to bovine teeth; the absence of interglobular dentin would not represent a significant difference according to what was observed in this study.

## INTRODUCCIÓN

Los dientes humanos son ideales para estudios *in vitro*, ya que proporcionan un excelente sustrato para evaluar las propiedades físicas, químicas y biológicas de los tejidos dentarios naturales, así como la respuesta de estos tejidos a materiales restauradores utilizados en terapia dental. Sin embargo, existen importantes limitaciones cuando se los utiliza en investigación: el bajo número de muestras, las superficies de trabajo curvas y pequeñas, y las consideraciones éticas que involucran su obtención. Por otro lado, la situación actual por la pandemia de COVID-19 y el tener que extremar las condiciones de bioseguridad, a fin de evitar contagios y propagación de enfermedades infecciosas, nos direcciona a buscar nuevas opciones en el campo de la investigación ^1^.

Los dientes bovinos han sido los más ampliamente reportados en la literatura dental, debido a algunas ventajas como la facilidad de obtener grandes cantidades de muestras, la amplia superficie de trabajo por su tamaño y la poca incidencia de caries que pueden afectar los resultados. Varios trabajos de investigación reportan similitudes en cuanto a la composición química del esmalte y la dentina, resistencia de los tejidos a aplicación de fuerzas, índices de refracción y mediciones cuantitativas ^2^^-^^5^.

El uso de dientes no humanos en la investigación odontológica ha planteado algunas preguntas por las diferencias en la composición y estructura de los tejidos dentarios. El criterio principal para la elección de un diente animal es que tenga semejanzas fisicoquímicas, estructurales y biológicas con el diente humano. Existen discrepancias o poca información respecto de las características histológicas de los dientes bovinos. 

Los seres humanos, en cuanto a su dentición, son del tipo bifodontos, es decir, presentan dos denticiones: una temporaria y otra permanente. Asimismo, son heterodontes, pues presentan diferentes formas anatómicas en sus grupos dentarios. Los bovinos son animales heterodontes y bifodontos de serie incompleta, ya que presentan formas y funciones diferentes, con incisivos planos y de borde cortante situados en el maxilar inferior. Se diferencian de los seres humanos en que no presentan grupo canino, pero sí grupos molares y premolares. El color de los dientes bovinos es similar al de los dientes humanos, pero su textura es distinta, ya que presentan estrías en sentido vertical sobre la superficie vestibular ^6^.

La sustitución de dientes humanos por dientes bovinos no es nueva y se ha recomendado para otros propósitos, como deposición de flúor y materiales dentales, incluyendo sistemas adhesivos, materiales de relleno del conducto radicular y procedimientos de blanqueamiento ^7^.

Los dientes bovinos presentan ventajas para su uso como sustitutos de dientes de humanos para la investigación de materiales dentales. Entre ellas encontramos que su manipulación es más sencilla, por ser de mayor tamaño; son fáciles de obtener, ya que, a diario, se sacrifican cientos de estos animales; la ausencia de caries, debido al tipo de dieta de estos mamíferos en su cría y reproducción, la cantidad de saliva y la cantidad de movimientos efectuados por la lengua; y, por último, su similitud tanto macroscópica como microscópica con los dientes humanos.

Este trabajo pretende contribuir al conocimiento respecto de las similitudes y diferencias de las características histológicas entre los dientes bovinos y humanos, lo que da soporte a otros estudios comparativos y promueve el uso de las piezas dentarias bovinas en trabajos de investigación en odontología.

## MATERIALES Y MÉTODOS

El diseño del estudio de tipo observacional y descriptivo, con utilización de unidades de análisis. Se utilizaron 20 piezas dentarias bovinas obtenidas de mataderos de la provincia de Corrientes, Argentina. Se realizó un muestreo no probabilístico por conveniencia en el cual se tuvieron en cuenta los siguientes criterios de inclusión: piezas dentarias bovinas permanentes sanas y con buen estado de fijación; y como criterios de exclusión se consideró las piezas dentarias incompletas o con alguna anomalía.

**Figura 1:**
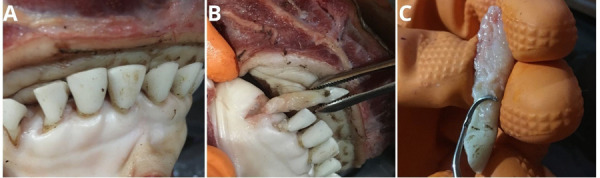
Proceso de obtención de muestras de dientes bovinos. En A se observa la pieza dentaria en el maxilar bovino. La figura B describe el retiro del alvéolo de la pieza dentaria luego del proceso de periostotomía, y los movimientos de divulsión dentaria para su extracción. En C se observa el curetaje y limpieza de la zona radicular para la eliminación del tejido periodontal.

Estas piezas se obtuvieron del maxilar del bovino, posrecambio dentario. Para su extracción, se tuvieron en cuenta las medidas de asepsia requeridas en la técnica para evitar cualquier tipo de contaminación (Fig. 1 ). El material se remitió en formaldehído buffer al 10% (Biopack, Argentina) al Servicio de Anatomía Patológica de la Facultad de Odontología, donde se aplicó la técnica histológica correspondiente. 

### Procesamiento de la muestra

Las piezas dentarias fueron colocadas en decalcificante de uso general para tejidos duros y ácido nítrico (Cicarelli, Argentina) al %7. Debido al tamaño de las piezas, se las colocó en decalcificante durante 14 días, el mismo que fue renovado a los 7 días verificando la marcha del proceso. La muestra fue seccionada mediante un bisturí quirúrgico de hoja N.° 12, el corte fue longitudinal en corona y raíz para permitir el ingreso del decalcificante.

Una vez decalcificadas totalmente, las piezas fueron lavadas con agua de grifo con sulfato de sodio al 5%, a fin de retirar cualquier residuo de decalcificante que pudiera interferir en los procesos posteriores ^8^.

Finalmente, se pasó al proceso de deshidratación para eliminar completamente el agua de la muestra tisular, y que así se pueda embeber adecuadamente en los medios de inclusión. Para ello se utilizó alcohol etílico (Biopack, Argentina) graduado en concentración creciente al 70%, 80% y 96%, durante 2 horas y moviéndolo cada 30 minutos. 

El aclaramiento se realizó con xilol (Cicarelli, Argentina), durante 1 hora con un cambio a los 30 min. Este procedimiento consistió en la sustitución del agente deshidratante por una sustancia miscible con el medio de inclusión que se utilizó, en este caso, parafina. 

La inclusión en parafina (56-58 °C *pellets*) se realizó mediante un baño en estufa de cultivo (Dalvo, Mod. MCM2, Argentina). El tiempo de cada baño varió por cada recipiente entre 1 hora y media a 3 horas. Para la conformación del bloque, el tiempo de cada baño varió según el tamaño y temperatura de la muestra. El bloque de parafina fue colocado en el micrótomo de deslizamiento y se obtuvo láminas con un espesor de aproximadamente 3 µm, las cuales fueron depositadas en un baño de flotación en una fuente de 15 cm de diámetro y agua a 20 °C, para finalmente ser alzadas a los portaobjetos de borde pulido (Exylon) y dejadas a secar aproximadamente de 12 a 24 horas a temperatura ambiente. 

### Tinción tricrómica de Masson

Para el proceso de desparafinización, se realizaron 4 baños de xilol de 10 a 15 minutos cada uno. Posteriormente, se realizaron sucesivos baños en alcohol etílico decreciente de 100°, 96° y 70°, durante 1 hora y 30 minutos.

La coloración se realizó con tinción especial tricrómica de Masson, del kit BIOPUR (Biopack-Argentina), pues esta permite diferenciar el núcleo celular, el citoplasma y las fibras de colágeno ^8^.

Para la coloración se siguieron las instrucciones del fabricante del kit: tinción con solución de trabajo de hematoxilina férrica de Weigert durante 10 min, lavado en agua de grifo durante 10 min, tinción con solución de Biebrich escarlata/fucsina ácida (3 min) (Rojo Masson), lavado con agua desmineralizada, tratamiento con solución de ácido fosfotúngstico al 2% (5 minutos), lavado con agua desmineralizada, tinción con solución de azul de anilina (2 minutos), lavado con agua desmineralizada, lavado en solución de ácido acético al 1% en agua desmineralizada (10 a 15 segundos) y deshidratación con alcohol con graduación creciente del 70%, 80% y 96%. Luego, el aclaramiento se realizó nuevamente con xilol, durante 1 hora con un cambio a los 30 minutos; y, finalmente, el montaje de los preparados histológicos se realizó con bálsamo de Canadá sintético (Biopack, Argentina) y cubreobjetos y portaobjetos de borde pulido.

Todos los preparados histológicos fueron observados y evaluados utilizando un microscopio binocular de luz (Zeisse Primostar, Argentina), con objetivos de diferentes magnificaciones (10X, 20X, 40X y 100X), para la descripción de los hallazgos histomorfológicos. 

### Consideraciones éticas 

El estudio fue aprobado por dictamen 151/20 del Comité de Bioética de la Facultad de Odontología de la Universidad Nacional del Nordeste, Argentina, y se tuvo en cuenta el anexo II de la Declaración de Helsinki, Principios éticos para las investigaciones en animales de laboratorio, de granja y obtenidos de la naturaleza.

## RESULTADOS

**Figura 2:**
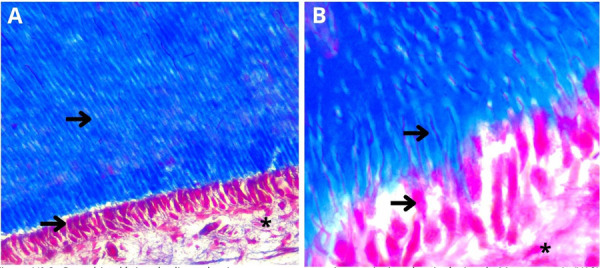
Corte histológico de diente bovino en zona coronaria con tinción tricrómica de Masson. En A (X10) se observa el complejo dentinopulpar. Las flechas indican la dentina con la presencia de túbulos dentinarios y la zona odontoblástica y subodontoblastica de la pulpa dental. En B (inmersión) se observa el complejo dentino-pulpar. Las flechas marcan la prolongación odontoblástica en el interior del túbulo dentinario y el núcleo de la célula odontoblástica. Los asteriscos (*) marcan núcleos de células subodontoblásticas.

En la observación microscópica panorámica del diente bovino, se pudo observar la ausencia del esmalte dental, debido a que este desaparece por la técnica histológica utilizada. Con relación al complejo dentino-pulpar, se observó la conformación clásica de pulpa dental, predentina y dentina. En la zona odontoblástica de la pulpa dental, se reconoce la organización en empalizada de los odontoblastos, los cuales se disponen con núcleos a diferentes alturas, sin seguir un patrón nuclear lineal (Fig. 2A).

En la zona odontoblástica, se pueden observar proyecciones citoplasmáticas de células que ingresan a la dentina, compatibles con las prolongaciones odontoblásticas que se encuentran alojadas en los túbulos dentinarios siguiendo un trayecto en “S” itálica característico. Dentro del túbulo dentinario y alrededor de la prolongación se observa un espacio libre que corresponde al espacio periprocesal (Fig. 2B).

La tinción tricrómica de Masson permitió observar la malla de fibras de colágeno, además de la íntima relación con el tejido pulpar. En la observación microscópica no halló la presencia de fibras colágenas dentro del espacio periprocesal, por lo que el componente fibrilar extracelular fue escaso a nivel dentinario. En lo que concierne a la zona subodontoblástica, mediante la coloración, se notó la existencia de fibras colágenas distribuidas en forma regular.

**Figura 3:**
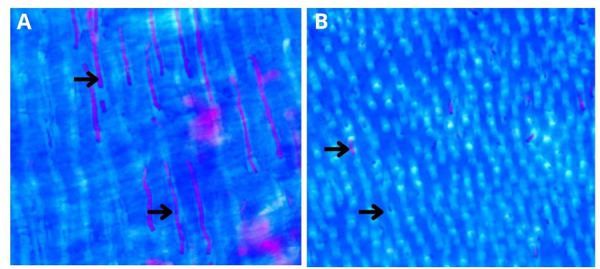
Corte histológico de diente bovino, con tinción de tricrómica de Masson, observación microscópica por inmersión, en A se observa la dentina en un corte longitudinal de los túbulos dentinarios, y en B un corte transversal, las flechas indican la prolongación odontoblástica dentro del tubulo dentinario, se observa tambien el espacio periprocesal, matriz intertubular y peritubular de la dentina bovina.

La dentina bovina presentó su histoarquitectura constituida por los túbulos dentinarios y la matriz intertubular y peritubular. En la observación, se encontraron los túbulos dentinarios en un corte transversal y, en su interior, el túbulo dentinario con la prolongación odontoblástica. Los túbulos dentinarios estaban limitados por dentina peritubular y, entre los anillos conformados por esta, se encontró dispuesta la dentina intertubular, que constituye el principal componente de la dentina circumpulpar del diente bovino (Fig. 3). 

La dentina peritubular puede ser diferenciada fácilmente de la dentina intertubular, debido a que presenta menos cantidad de fibrillas de colágeno y mayor proporción de proteoglicanos sulfatados, mientras que la dentina intertubular contiene gran cantidad de colágeno.

**Figura 4:**
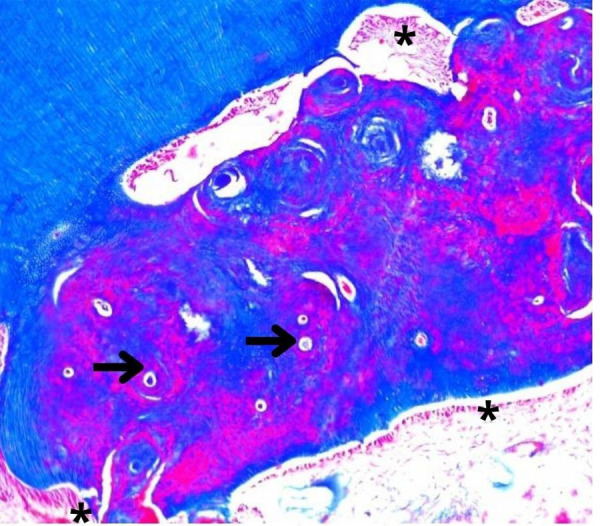
Corte histológico de diente bovino con tinción tricrómica de Masson. En A (X10) se observan zonas de dentina dispuesta de forma irregular. Las flechas indican prolongaciones odontoblásticas únicas con depósito dentinario periférico. Los asteriscos (*) indican zonas donde se encuentran los odontoblastos en forma de empalizada.

En otras zonas se observó dentina de forma desorganizada, que no siguió el patrón morfológico regular característico de túbulos de forma en “S” itálica de los dientes humanos. En este caso, se puede observar el túbulo dentinario, el espacio peritubular y, en su interior, la prolongación odontoblástica. Periféricamente, se halló el depósito concéntrico de dentina como capas de tejido que pueden referir periodos de actividad y de descanso en la formación de dentina. No se observó dentina interglobular (Fig. 4).

## DISCUSIÓN

Cuando se observan los dientes bovinos mediante microscopía óptica, se observa que la dentina bovina, al igual que la de los humanos, está formada principalmente por los túbulos dentinarios y una matriz intertubular ^9^^-^^12^. Díaz ^12^ y Romero^11^, en sus tesis, describieron los túbulos dentinarios que, al atravesar todo el espesor de la dentina, siguen un trayecto en forma de “S” levemente acentuada, desde la unión amelodentinaria hasta la pulpa, lo que coincide con lo observado en este estudio. 

Estas investigaciones también coinciden con Fuentes ^13^, quien refiere la conformación de la dentina humana en una base tubular y una matriz intertubular formada por colágeno de tipo I. 

Los túbulos dentinarios del diente bovino se extienden por todo el espesor de la dentina, desde la pulpa hasta la unión amelodentinaria o cemento dentinario, en coincidencia con lo expuesto por Alano *et al*. ^14^ cuando se refieren a la dentina humana.

Ortiz y Salazar ^15^ realizaron estudios histológicos en pulpas dentales de ratones con tinción tricrómica de Masson, y observaron la escasez de matriz extracelular fibrilar a nivel del espacio periprocesal. Resultados similares se observaron en los dientes bovinos analizados en este estudio.

Por el gran tamaño de los dientes de bovino, el diámetro y la cantidad de los túbulos dentinarios son mayores que en los dientes humanos, especialmente en la dentina radicular. Esta característica coincide con los datos de trabajos realizados por Peláez-Vargas ^16^, los mismos que, por otro lado, tampoco encontraron zonas de dentina interglobular.

Schilke *et al*. ^17^ compararon el número y diámetro de los túbulos entre dientes de bovino y humanos, sin encontrar diferencias significativas en el tamaño al comparar los túbulos dentinarios, así como en el número de túbulos en lo que corresponde a dentina radicular y coronaria. Estos resultados difieren de los encontrados en este estudio y los de Peláez y Vargas ^16^.

En la observación microscópica de 100x por inmersión, se observó la presencia de dentina peritubular e intertubular, y la prolongación odontoblástica en el interior del túbulo dentinario. Por otro lado, en las zonas externas de la dentina, los túbulos ya no se observan tan definidos y no presentaron prolongaciones odontoblásticas en su interior.

Del mismo modo, los resultados en cuanto a número y disposición de los túbulos dentinarios en el espesor de la dentina, entre el límite amelodentinario o cemento dentinario y la pulpa dental fueron similares a los descritos por Fuentes en trabajos sobre dentina humana ^13^.

Midwar ^18^ refiere que los dientes humanos son similares morfológica e histológicamente a los dientes bovinos, lo cual coincide con los resultados obtenidos en este estudio.

Estudios realizados mediante microscopía electrónica y comparando micromorfología y microdureza tanto de la dentina bovina como la humana, demostraron que la dentina esclerótica bovina presenta un mayor grado de microdureza que la humana, lo que debe tenerse en cuenta para estudios de adhesión en dientes bovinos ^(^^19^^-^^30^.

Limitaciones: La posibilidad de una mayor generalización de estos resultados está limitada. Más estudios con otros métodos de evaluación de la dentina deben ser realizados para confirmar los resultados de esta investigación. 

## CONCLUSIÓN

La dentina bovina puede ser considerada como un material de estudio propicio para trabajos de investigación odontológica in vitro, ya que, al realizar el análisis histomorfológico, se observa similar a la dentina humana. Adicionalmente, las piezas dentarias bovinas son de fácil obtención y presentan un tamaño ideal para su manipulación. La ausencia de dentina interglobular no representaría una diferencia significativa para su utilización.
